# Factors associated with the impact of fixed appliance orthodontic treatment on the oral health-related quality of life of adolescents: Assessment using a condition-specific instrument

**DOI:** 10.4317/jced.61437

**Published:** 2024-04-01

**Authors:** Larissa Corradi-Dias, Saul-Martins Paiva, José-Alcides-Almeida de Arruda, Gabriela-Luiza-Nunes Souza, Rodrigo-Keigo Nakagawa, Alexandre-Fortes Drummond, Leonardo-Foresti-Soares de Menezes, Lucas-Guimarães Abreu

**Affiliations:** 1 Department of Child and Adolescent Oral Health, School of Dentistry, Universidade Federal de Minas Gerais, Belo Horizonte, Minas Gerais, Brazil; 2 Department of Oral Diagnosis and Pathology, School of Dentistry, Universidade Federal do Rio de Janeiro, Rio de Janeiro, Brazil; 3 Department of Restorative Dentistry, School of Dentistry, Universidade Federal de Minas Gerais, Belo Horizonte, Minas Gerais, Brazil

## Abstract

**Background:**

Oral outcomes may have an impact on quality of life. The aim of this study was to assess factors associated with the impact of fixed appliance orthodontic treatment on the oral health-related quality of life (OHRQoL) of adolescents.

**Material and Methods:**

Individuals aged 10 to 18 years undergoing orthodontic treatment were included. Data regarding adolescents’ sex and age, parental schooling, family income, and number of individuals who depend on income were collected. Clinical variables, orthodontic tooth extraction and malocclusion severity were also analyzed. OHRQoL was assessed with a questionnaire with items distributed across nine domains: aesthetics, functional limitation, diet, hygiene, maintenance, physical impact, social impact, time constraints, and transport/cost inconveniences. The higher the score, the more negative the adolescent’s perception of his/her OHRQoL. Statistical analysis was performed.

**Results:**

Seventy-five adolescents participated. Individuals aged ≤12 years had a more negative perception of the diet domain (*p*=0.026). Individuals whose parents/guardians had ≥8 years of schooling had a more negative perception of the impact on the hygiene domain (*p*<0.024). Individuals whose families had an income of ≤2 salaries had a more negative perception of the maintenance domain (*p*=0.016). Girls had a more negative perception of the physical impact domain (*p*<0.018). Girls (*p*=0.011), adolescents whose families had an income of ≤2 salaries (*p*=0.003), and adolescents who had severe malocclusion (*p*=0.026) had a more negative perception of the transport/cost inconveniences domain. Girls had a significantly higher overall score in response to the questionnaire than boys (*p*=0.041).

**Conclusions:**

Adolescents’ sex, age, and malocclusion as well as parental education and family income were associated with the impact of orthodontic treatment on adolescents’ OHRQoL.

** Key words:**Adolescent, Quality of life, Orthodontic treatment, Fixed appliance.

## Introduction

Malocclusion is defined as an abnormal position of the teeth or an altered relationship between the maxilla and the mandible, leading to an appreciable deviation from ideal occlusion that causes aesthetic and functional changes to the affected individual (1). The physical appearance of a person (e.g., dentofacial traits) is an important aspect of perception and positioning of this individual in relation to his/her peers (2). An individual’s view of functional aspects, as well as emotional and social well-being, encompasses the construct of the oral health-related quality of life (OHRQoL) of a person (3).

The literature has endorsed that such construct among adolescents is deeply influenced by the individual’s sex and age, as well as by socioeconomic conditions, severity of malocclusion, and other associated factors (4-6). Orthodontic treatment aims to correct dental and skeletal changes, allowing the individual to have a more favorable occlusion in terms of aesthetic and functional features (1). On this basis, the seeking of and adherence to orthodontic treatment are influenced by the individual’s desire to obtain a more harmonious facial appearance and, consequently, to improve aspects of his/her well-being (2). Nevertheless, during orthodontic treatment, variations in the OHRQoL are observed among adolescents. For instance, a recent study demonstrated that girls have a more negative perception of the wearing of fixed appliances than boys. This exacerbated negative perception is mainly influenced by physical and social issues related to the wearing of an orthodontic device (7). Another study employing a generic quality of life questionnaire to assess adolescents undergoing orthodontic treatment reported that individuals whose families had a worse socioeconomic status had a more negative perception of OHRQoL than their peers whose families had more favorable socioeconomic conditions (5). Thus, several factors seem to be linked with the impact of fixed appliance wearing on the OHRQoL of individuals (5,7).

Validated instruments evaluating the impact of oral conditions on the OHRQoL of adolescents have been reported elsewhere (8). Nevertheless, these instruments assess the impact of oral conditions in general and may, indeed, not be responsive or faithful when used to evaluate the impact of a very specific condition such as the wearing of fixed appliances (9). In 2006, in the United Kingdom, authors developed the Impact of Fixed Appliance Measure (IFAM), a condition-specific instrument used to assess the impact of fixed appliances wearing on the OHRQoL of adolescents (10). This instrument was translated, cross-culturally adapted for use in the Brazilian population and named B-IFAM (11). This questionnaire is considered to provide more reliable results on the real impact of the wearing of fixed appliances on the OHRQoL of young individuals (7,10,11). Therefore, the present study aimed to evaluate the factors associated with the perception of Brazilian adolescents regarding the impact of orthodontic treatment with fixed appliances on the OHRQoL, using the B-IFAM tool.

## Material and Methods

-Study design and ethical issues

The checklist Strengthening the Reporting of Observational Studies in Epidemiology (STROBE) (12) was used as a template for the reporting of this longitudinal study. The study was approved by the Ethics Committee of Universidade Federal de Minas Gerais (UFMG) (No. 62116216.2.0000.5149). Participant anonymity was guaranteed according to the Declaration of Helsinki.

-Participants, setting, and data collection period

The sample of this study consisted of 80 individuals. Male and female adolescents aged 10 to 18 years, who were beginning orthodontic treatment with fixed appliances at the School of Dentistry of UFMG, in Belo Horizonte, Brazil were included in this study. Excluded were adolescents with cognitive disorders reported by parents/caregivers or those with craniofacial anomalies/disorders. Participants’ recruitment was performed between January 2017 and February 2020.

-Data collection

Instrument for OHRQoL evaluation

The impact of orthodontic treatment with fixed appliance on adolescents’ quality of life was evaluated with the B-IFAM. This instrument was developed in England (10), translated, cross-culturally adapted, and validated for use on Brazilian adolescents between 10 and 18 years (11). The B-IFAM has 43 items distributed across nine domains: aesthetic impact (five items), functional impact (three items), dietary impact (six items), oral hygiene impact (three items), maintenance impact (two items), physical impact (nine items), social impact (five items), time constraints (five items), and travel/cost/inconvenience impact (five questions). For each item, the response options are scored on a Likert scale ranging from 1 to 5 as follows: 1, strongly disagree; 2, disagree; 3, neither agree nor disagree; 4, agree, and 5, strongly agree. The overall B-IFAM score ranges from 43 to 215. The higher the score, the more negative is the perception of the adolescent regarding the impact of the wearing of fixed appliances on his/her OHRQoL. Scores for the domains are also possible and the rationale for interpretation is similar to that of the overall score (10,11).

Each adolescent filled out the B-IFAM at three times after the bonding of fixed appliances: T1, 1 month after bonding; T2, 3 months after bonding, and T3, 6 months after bonding. Parents/caregivers assisted the adolescents when answering the items of the last two domains, according to a previously published method (10,11).

Clinical examination

For the evaluation of the severity of malocclusion and the indication of the orthodontic extraction of premolars (yes/no), the study subjects were clinically examined by a dentist (L.C.D.) who had been previously calibrated. The clinical examination was conducted in a separate clinic with dental equipment (chair and supplies), under artificial light, and using a World Health Organization probe and clinical mirror.

The Dental Aesthetic Index (DAI) was employed for the assessment of the severity of malocclusion. The DAI is an index with which 10 occlusal characteristics are evaluated: number of missing anterior teeth, crowding in the anterior region of the maxilla and mandible, spacing in the anterior region of the maxilla and mandible, diastema between the upper central incisors, the worst irregularity in the anterior region of the maxilla and of the mandible, overjet, anterior crossbite, open bite, and the relationship between the upper and lower first molars. The scores attributed to each characteristic are multiplied by a coefficient and summed to the constant 13 in order to obtain the overall DAI score (13). Based on the overall DAI score, the adolescents were assigned to the following subgroups: DAI≤25: slight malocclusion, DAI-26-30: defined malocclusion, and DAI≥31: severe or very severe malocclusion.

Demographic and socioeconomic data

Demographic and socioeconomic data were collected by means of an interview with the adolescents’ parents/caregivers. The following information was collected: adolescents’ sex and age, parents’/caregivers’ schooling (<8 years of study/≥8 years of study), monthly family income (<2 minimum wages/≥2 minimum wages), and number of individuals in the household who depend on the income (<3 individuals/≥3 individuals). For parents’ schooling, the highest number of years of study between the father and the mother of the adolescent was registered.

-Data analysis

The Statistical Package for the Social Sciences (SPSS) software (IBM SPSS Statistics for Windows, version 23.0: IBM Corp.) was used for statistical analysis of the data. Descriptive analysis was conducted. The association of the variables analyzed in this study (clinical, demographic, and socioeconomic characteristics, as well as time of orthodontic treatment) with the OHRQoL of adolescents undergoing orthodontic treatment with fixed appliances was evaluated by Analysis of Covariance (ANCOVA). The level of significance was set at *p*<0.05 in all analyses.

## Results

**Figure 1:**
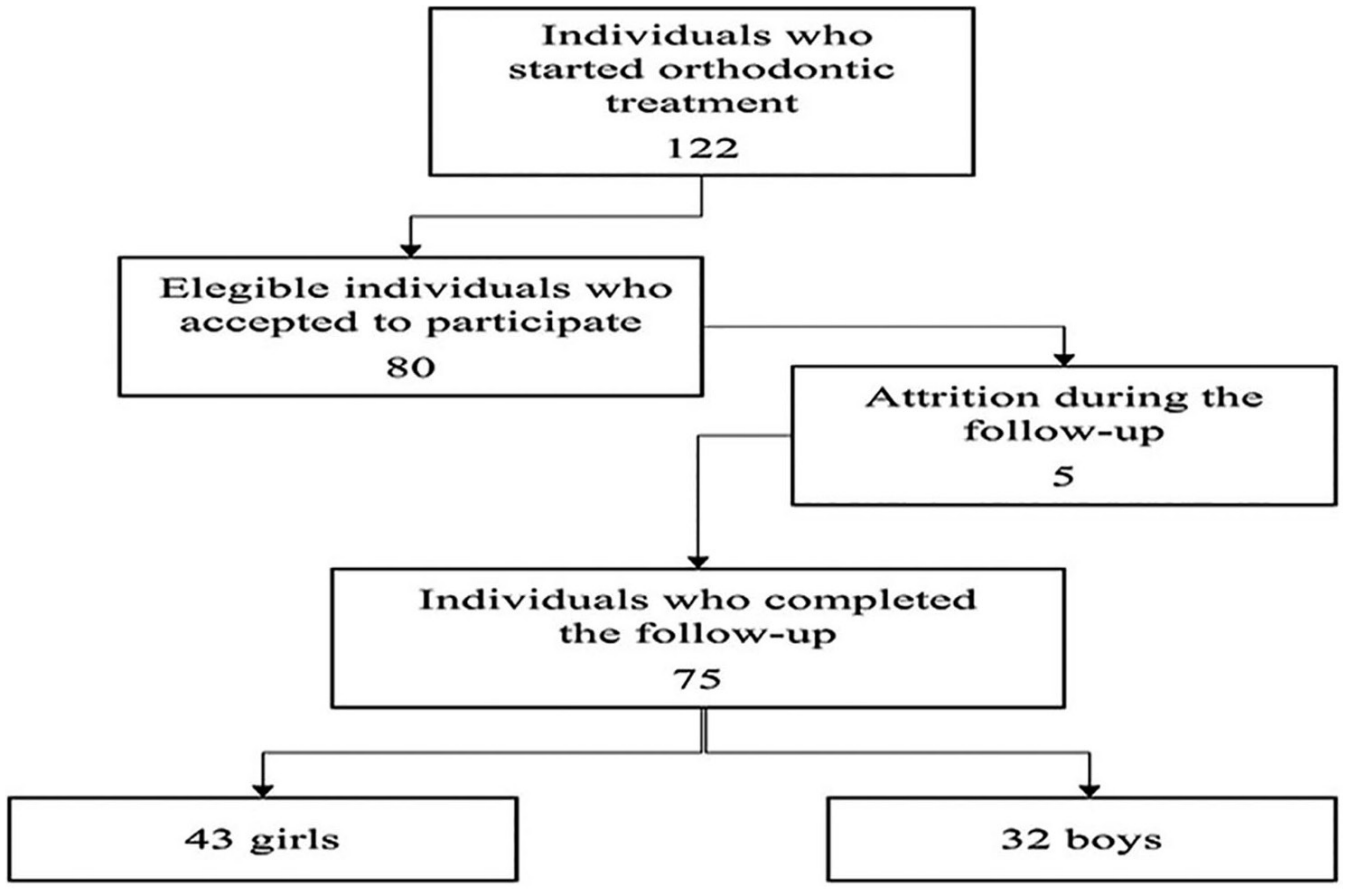
Flowchart of the study.

Among the 80 adolescents who had started follow-up, five were excluded due to missing data. Of the 75 who participated in the entire follow-up, 43 (57.3%) were girls and 32 (42.7%) were boys. Mean age was 12.4 years (±1.79). Figure 1 shows the flowchart of the study.

Regarding the severity of malocclusion, 19 (25.3%) exhibited slight malocclusion, 24 (32.0%) exhibited defined malocclusion, and 32 (42.7%) exhibited severe or very severe malocclusion (Table 1). No difference was observed between adolescents who participated in the entire follow-up and excluded adolescents regarding the following variables: adolescents’ sex and age, parents’/caregivers’ schooling, monthly family income, number of individuals in the household who depend on the income, indication of orthodontic extraction, and severity of adolescents’ malocclusion (*p*>0.05) (Table 2).

**Table 1:**
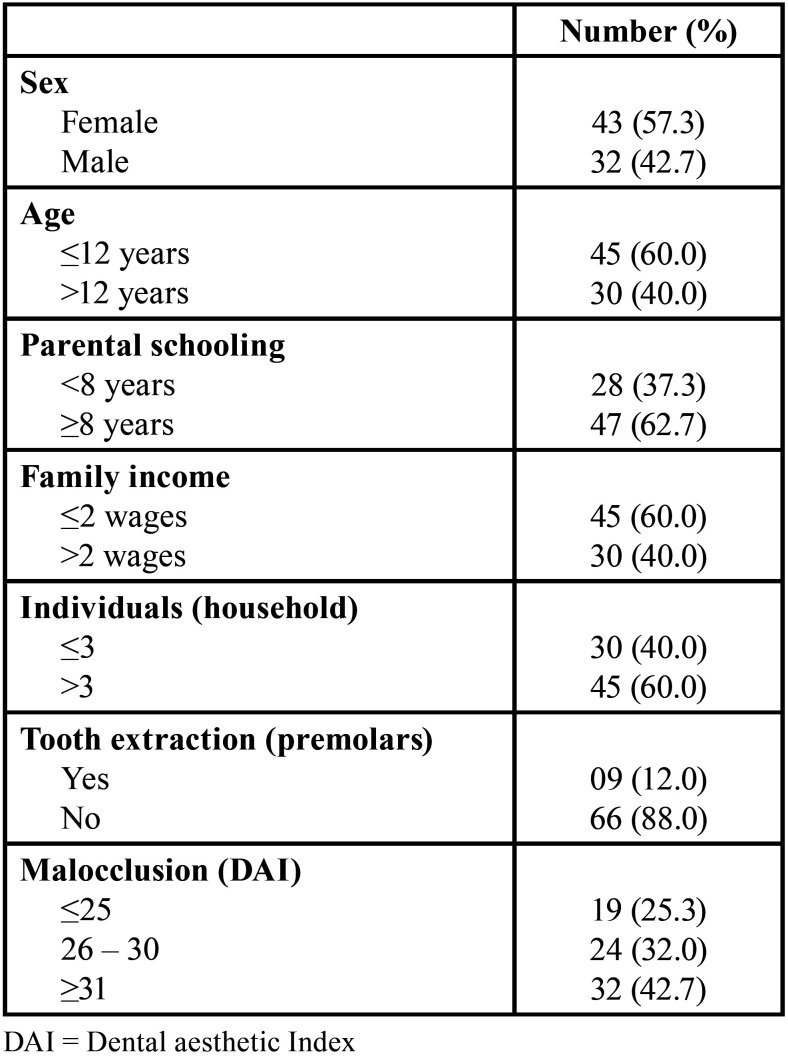
Sociodemographic characteristics and orthodontic treatment need of the participants.

**Table 2:**
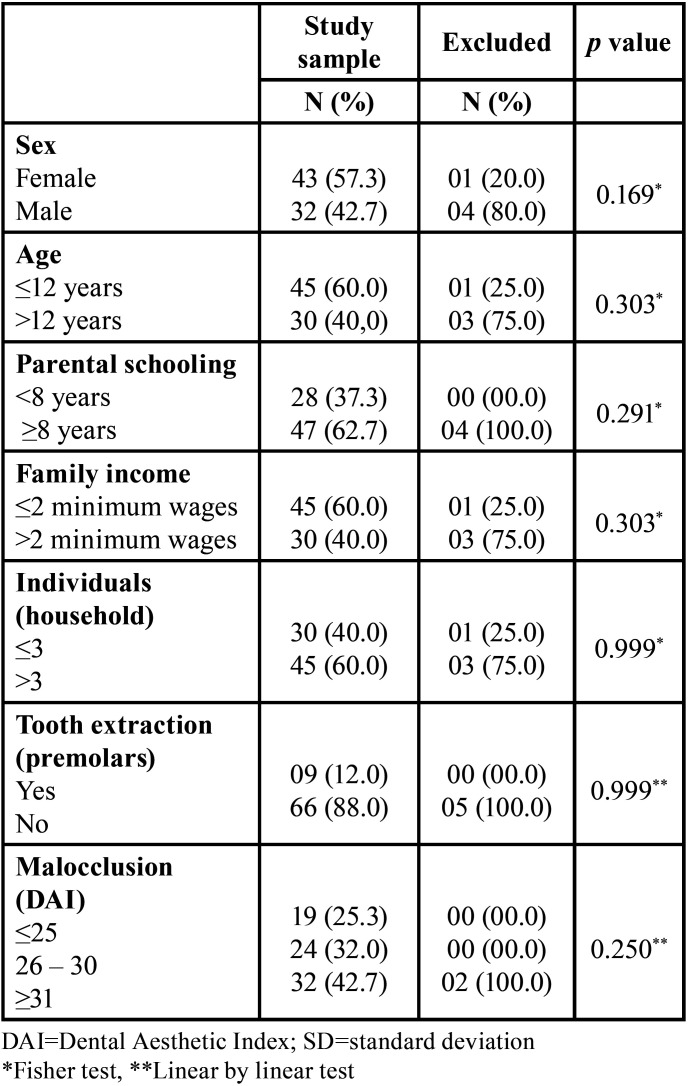
Comparison of adolescents in the study sample with those excluded because of missing values.

Adolescents aged ≤12 years had a significantly higher score in the diet impact domain than adolescents aged >12 years (*p*=0.026). Adolescents whose parents/caregivers had ≥8 years of study exhibited a significantly higher score in the oral hygiene impact domain than adolescents whose parents/caregivers had <8 years of study (*p*=0.024). Adolescents whose families had an income of ≤2 minimum wages exhibited a significantly higher score in the maintenance impact domain than adolescents whose families had an income of >2 minimum wages (*p*=0.016) (Table 3).

**Table 3:**
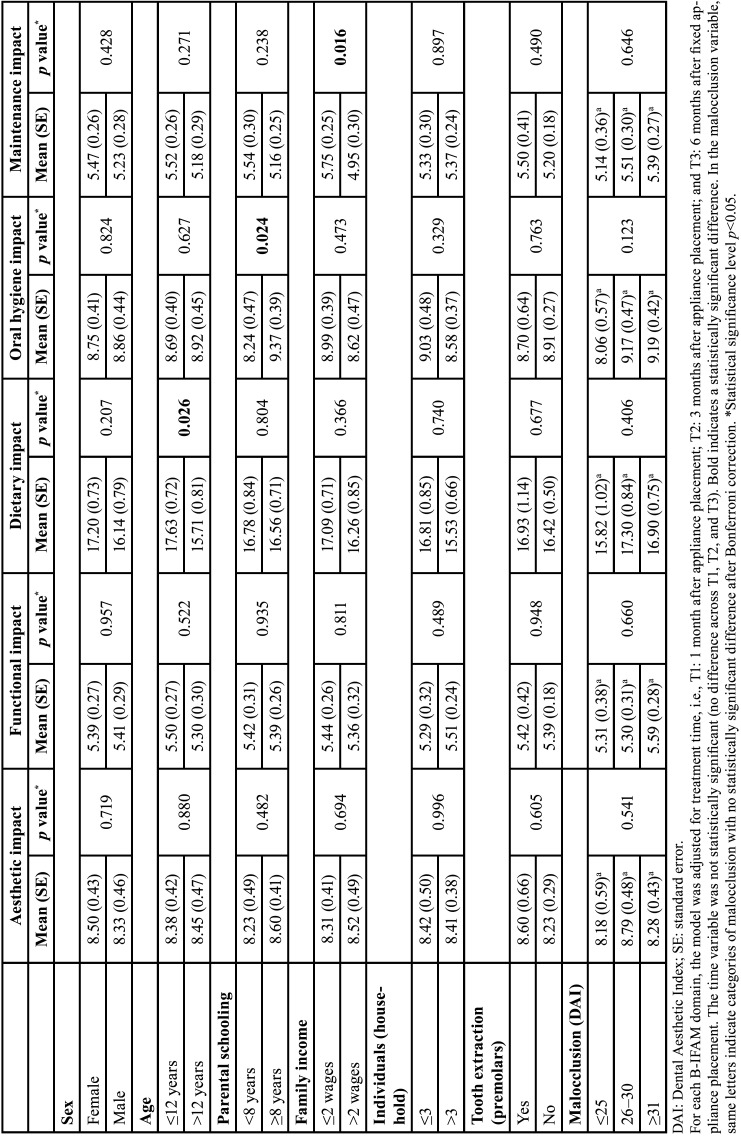
Evaluation of the association of factors related to the quality of life of individuals undergoing orthodontic treatment with the aesthetic, functional, dietary, oral hygiene, and maintenance impacts.

Girls had a significantly higher score in the physical impact domain than boys (*p*=0.018). Girls, adolescents whose families had an income of ≤2 minimum wages, and adolescents with severe or very severe malocclusion exhibited a significantly higher score in the transport/cost/inconvenience domain than boys (*p*=0.011), adolescents whose families had an income of >2 minimum wages (*p*=0.003), and adolescents with slight malocclusion (*p*=0.026). Girls had a significantly higher overall B-IFAM score than boys (*p*=0.041) (Table 4).

**Table 4:**
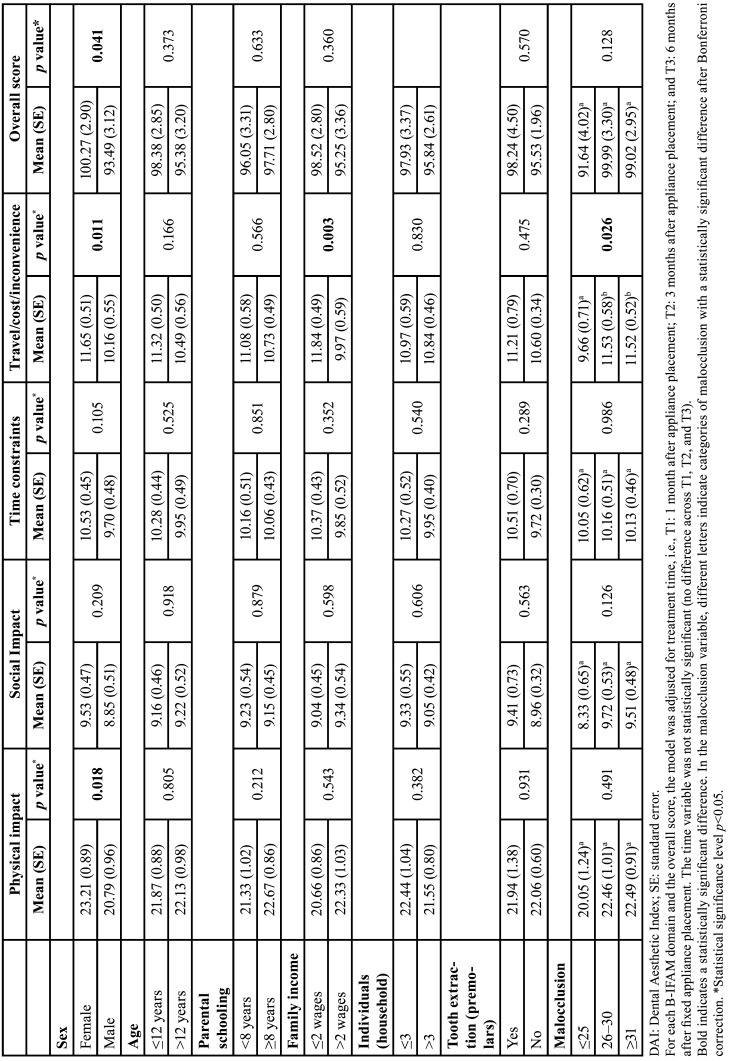
Evaluation of the association of factors related to the quality of life of individuals undergoing orthodontic treatment with the physical impact, social impact, time constraints, travel/costs/inconvenience, and overall score of the Brazilian version of the Impact of Fixed Appliances Measure (B-IFAM).

## Discussion

The purpose of the present study was to explore factors associated with the impact of orthodontic treatment with fixed appliances on the quality of life of adolescents using a specific-condition questionnaire, namely B-IFAM. We noticed that the impact on diet was more negative among younger adolescents aged ≤12 years. In the B-IFAM, questions in the diet impact domain are related to how much individuals miss foods that should be avoided while wearing fixed appliances. In particular, these results may be related to the difficulties of very young individuals in following the orthodontist’s recommendations to avoid certain types of foods during the course of orthodontic therapy. During the wearing of fixed appliances, hard, sticky, and high sugar foods should be avoided since they can break or damage the wires and brackets (14), or even contribute to the development of dental caries (15). In this respect, younger adolescents should definitely be counseled since they tend to consume excessively sugary foods and drinks, especially in the form of sugar sweetened beverages (16). These individuals may need greater support from orthodontists and parents/guardians regarding the diet for a successful treatment, without complications or breakage of the orthodontic devices (17).

In the oral hygiene domain, the impact was more negative among adolescents whose parents had a higher educational level. Parents with a higher level of schooling may be more concerned about their children brushing their teeth and thus may realize that the performance of oral hygiene during the wearing of fixed appliances is more difficult (18). Thus, this finding may indicate to orthodontists that adolescents and their parents/guardians with lower educational levels need to receive reinforced instructions about oral hygiene during orthodontic therapy since this does not seem to be a major concern for these individuals. On this basis, it is also important to note that significant worldwide disparities are observed regarding epidemiological indicators of tooth brushing among adolescents. The estimated overall prevalence is that 8.6% of adolescents aged 12 to 15 years in low- and middle-income countries never brush their teeth, while 80.9% of them routinely brush their teeth once to three times a day, and 9.7% more than three times a day (19). In Brazil, for instance, some private and public insurance companies cover orthodontic treatments, although to a limited extent. Thus, individual oral hygiene habits may differ according to the type of orthodontic therapy or the sociocultural aspects of the adolescents and their parents/guardians (20).

In our study, girls had more negative perceptions of physical impact than boys. Other studies that compared the side effects of orthodontic therapy found that adolescent girls reported greater overall pain intensity, pain when eating, and discomfort in their daily routine caused by the wearing of the fixed device compared to adolescent boys (7,21). Accordingly, a former study reported that girls aged 8 to 12 years were found to be more dental fearful than boys. The authors claimed that this association is due to cultural issues, with girls being likely to feel more comforTable to express their feelings and confess their fears (22). Physiologically, it is virtually unknown why males and females do not experience pain in the same way. A spectrum of characteristics, including genetics, anatomical development, and hormone levels, each of which may affect a person’s needs in pain therapy, cannot be ruled out (23). Adolescents who wear fixed orthodontic appliances are more likely to feel a higher negative impact on their OHRQoL than those who do not wear such appliances (24). Thus, we emphasize that the orthodontist should be aware of the fact that, when advising and guiding adolescents about the adverse effects of fixed appliances, girls may need more support and oral care to realize that pain and discomfort can be temporary impairments during therapy for the correction of malocclusion (21). Discomfort after consultations for activation of the fixed appliance should be considered a possible event (25) and anticipatory guidance from the orthodontists can mitigate the complaints of adolescents about orthodontic treatment (26).

Adolescent girls from lower income families and with more severe malocclusion had a more negative perception of the impact of the transport/cost inconveniences domain. In general, parents/caregivers of low-income families, when deciding about the orthodontic treatment of their child, may face difficulties in bearing the costs of the therapy and additional costs during the course of treatment (27). These costs usually involve transportation for monthly visits to activate the fixed appliances or are related to orthodontic mechanics, which sometimes require ancillary orthodontic devices and a longer time to correct a more severe malocclusion (28). These issues of treatment costs may also have been determinants for the greater negative impact on the maintenance impact domain among young people from lower income families. In fact, the concern of the adolescents and their families about honoring their financial commitment to the orthodontist at the time of breakage of orthodontic devices and brackets debonding may have exacerbated the frustration of young patients, especially girls. These complications were perceived as possible causes of the failure of the orthodontic therapy they were undergoing, or at least of a longer and more costly treatment due to accidents with the components of the appliance (29).

A more negative impact on girls in the transport/cost inconveniences domain as well as in the physical impact domain may have been a contributory factor to an exacerbated negative impact on their overall B-IFAM score compared to their male peers. In this scenario, the results of this study might be very useful for orthodontists who devote their time to the provision of orthodontic services for adolescents. These professionals should be aware of factors related to the impact that the wearing of fixed appliances can have on adolescents undergoing orthodontic therapy, so that they can counsel the young adolescents and their parents/guardians about certain adversities that may appear during orthodontic treatment (30). An orthodontist who is aware of these details (patients’ perceptions of the treatment) and who offers support to the individual wearing fixed appliances and his/her family can help increase the level of patient cooperation with treatment, increasing the chances of success (31). Guided by the orthodontist, these individuals may find that, even in the presence of some inconveniences caused by the wearing of orthodontic braces (e.g., discomfort, difficulty in performing some functions, problems during feeding, oral hygiene, and treatment costs), they are moving towards the correction of malocclusion and a better dentofacial appearance (32).

The limitations of this study include the absence of an assessment of OHRQoL before fixed appliance placement (i.e., baseline) since the tool employed here was a specific questionnaire for the evaluation of individuals wearing fixed appliances. The questionnaire was applied on the day of activation of the fixed orthodontic appliance; the perception of the different phases of treatment, therefore, was not evaluated. Furthermore, since the perception of quality of life is expected to depend on the culture of the individual, the magnitude of wearing an orthodontic appliance may vary across the country where the study was conducted. Thus, future studies deserve a cross-cultural assessment of the impact of fixed appliances among adolescents from different Brazilian regions in a multicenter investigation.

Adolescents usually seek orthodontic treatment to correct malocclusion and improve their dental and facial aesthetics. Awareness of the factors that influence individuals’ oral health-related quality of life during fixed appliance therapy is crucial for orthodontists, dentists, dental hygienists, and other healthcare professionals. This study can help identify which specific demographic and clinical characteristics of patients, have the most substantial impact on an adolescent’s overall well-being. By identifying these factors, clinicians can tailor their treatment plans and provide better patient support, ultimately enhancing patient experience and outcomes. Additionally, the findings of this study can inform the development of guidelines and strategies to mitigate potential adverse impacts and maximize the benefits of orthodontic treatment, leading to improved oral health and overall quality of life for adolescents undergoing orthodontic treatment.

In summary, multiple factors such as sex, age, malocclusion, parental schooling, and family income were associated with the impact of fixed appliance orthodontic treatment on adolescents’ quality of life.
